# COVID-19, Wellness and Life Satisfaction in Adolescence: Individual and Contextual Issues

**DOI:** 10.3390/ijerph20085600

**Published:** 2023-04-20

**Authors:** Margarida Gaspar de Matos, Marina Carvalho, Cátia Branquinho, Catarina Noronha, Bárbara Moraes, Tania Gaspar, Fábio Botelho Guedes, Ana Cerqueira, Osvaldo Santos, Nuno Neto Rodrigues

**Affiliations:** 1 ISAMB/Aventura Social, Lisbon University, 1649-026 Lisbon, Portugal; 2 APPSICY, ISPA University Institute, 1149-041 Lisbon, Portugal; 3 Faculty of Human Sciences, Portuguese Catholic University, 1649-023 Lisbon, Portugal; 4 Manuel Teixeira Gomes Higher Institute, Algarve University Hospital Centre, 8500-508 Portimão, Portugal; 5 HEI-Lab, Lusófona University, 1749-024 Lisbon, Portugal; 6 Directorate General of Education and Science Statistics, 1399-054 Lisbon, Portugal

**Keywords:** wellness, life satisfaction, adolescence, COVID-19

## Abstract

During and in the aftermath of the COVID-19 pandemic, several works reflected on young people’s physical and psychological health. The Dual Factor Model, which we refer to as the quadripartite model, is useful for understanding children’s and adolescents’ psychological health and differentiating them regarding their attitude toward the effects of the COVID-19 pandemic. In this investigation, students from the fifth to twelfth year of schooling enrolled in the DGEEC study “Psychological Health and Wellbeing in Portuguese schools” were considered. Four groups were created based on life satisfaction (low or high) and psychological distress (with or without symptoms). The study included 4444 students (M = 13.39 years ± 2.41), of whom 47.8% were male. Of the participants, 27.2% were in the second cycle of primary education, and 72.8% were in lower and upper secondary education. Differences in gender and education level (as a proxy for age) were observed. Additionally, when considering students’ perceptions of changes in their lives following the COVID-19 pandemic (stayed the same, became worse, became better), these three groups were compared concerning personal and contextual variables, revealing significant differences at both the individual and contextual levels. Finally, the study discusses the influence of education and health professionals and the need for friendly public policies.

## 1. Introduction

Several studies have reflected on the situation of young people, particularly their physical and psychological health associated with the COVID-19 pandemic [1,2,3,4,5]. There is a consensus in the literature regarding the psychosocial impacts of the pandemic among school-age children and adolescents [6].

Tomé and colleagues point out that young people’s mental health, wellbeing and life satisfaction are influenced by social engagement [7]. Therefore, given the contingencies grounded in the COVID-19 pandemic (more specifically, social distancing and general confinement of the population), young people’s psychosocial contexts are considered to have undergone changes that, in turn, generated risks for their psychological health and life satisfaction [8,9,10,11,12].

In a qualitative study by Branquinho and Matos, adolescents reported increased depressive and anxiety symptoms and increased perceptions of loneliness [13]. Quantitative studies across Europe corroborate this association, identifying an increase in depression, anxiety, and stress symptoms [9,10,14,15].

According to UNESCO, government measures in response to the COVID-19 pandemic have affected more than 90% of young individuals [16]. The confinement measures implemented due to the pandemic were anticipated to have a negative impact on the physical and mental wellbeing of young people [4,8,17,18,19], as well as their academic performance [20] and health-related behaviors such as sleep, diet, and physical activity [12,17]. Due to COVID-19 restrictions, adolescents had to remain at home, resulting in decreased physical activity and reduced opportunities to socialize with peers, ultimately leading to social isolation [17,21]. In addition, anxiogenic factors emerged, such as the fear of contracting the virus [22] and the risk of exposure to adverse events in family contexts (violence); furthermore, other challenges became inevitable due to an increased lack of access to essential resources and economic difficulties [1,2,3,4,22]. 

The dual factor model [23,24,25], which we refer to as the quadripartite model for its use in population studies, highlighted that life satisfaction and psychological symptoms are not opposed in a continuum. Instead, the two variables are distinct dimensions, respectively linked to psychological health and psychological distress. These dimensions define four psychological states: Complete Psychological Health (indicating reduced psychological symptoms and high life satisfaction), Incomplete Psychological Health (indicating reduced psychological symptoms and low life satisfaction), Incomplete Psychological Distress (indicating marked psychological symptoms and high life satisfaction), and Complete Psychological Distress (indicating marked psychological symptoms and low life satisfaction). The dual factor model [23,24,25] was thus the theoretical frame for the current analysis, assuming four situations instead of a linear continuum of wellbeing and mental distress.

The main objective of this study was to examine how second cycle and lower and upper education pupils perceive changes in their daily lives due to the COVID-19 pandemic, using the dual factor model as a theoretical framework. This model identifies four distinct groups that range from complete psychological distress to complete psychological health, based on the combination of high or low life satisfaction and reduced or marked psychological symptoms. In addition, the study sought to investigate differences in various individual and contextual factors associated with pupils’ perceptions of changes in their daily lives related to COVID-19.

## 2. Method

### 2.1. Procedure

The Psychological Health and Wellbeing|School study was a joint effort between the Directorate General of Education and Science Statistics, the Directorate General of Education, the National Programme for the Promotion of School Success, Aventura Social Team/ISAMB, the University of Lisbon, the Order of Portuguese Psychologists, and the Calouste Gulbenkian Foundation. This study, which had the Ministry of Education’s approval, began in December 2021.

Sampling used a stratified and random selection of public schools in Mainland Portugal by geographic region. The liaison teachers and psychologists of the participating schools administered the data collection instruments in the respective computer rooms in February and March 2022 after a stratified and random selection of classes from each school group. Only students with parental consent and acceptance of informed consent completed the online questionnaires. The average duration of the application protocol was between 20 and 30 min. The procedures and outcomes are described in detail in the study report [3], available online.

### 2.2. Participants 

This study included 4444 students (M = 13.39 years ± 2.414; Min = 9 and Max = 18), of which 47.8% were male and 52.2% female. Of the participants, 27.2% were in the second cycle of primary education, and 72.8% were in lower and upper secondary education (Table 1).

### 2.3. Measures

The present work considered the evaluation protocols for the second cycle and lower and upper secondary education. The measures and variables under study are described in Table 2.

### 2.4. Data Analysis

Data were analyzed using SPSS 25.0 (SPSS, Chicago, IL, USA). First, chi-square analyses were performed to check the distribution of pupils by gender and cycle/level of education age proxy according to each of the four situations based on the dual factor model. Furthermore, single-factor analyses of variance were performed, with subsequent multiple comparison analyses using Tukey’s method. In each analysis, the basic assumptions for this type of procedure were considered (n and group size, normality and homogeneity of variances). In all analyses, a confidence level of 95% was considered. 

## 3. Results

Table 3 shows the distribution of frequencies of these pupils for each of the main variables under study: life satisfaction and psychological symptoms.

Two groups were created from the measurement of life satisfaction, and the other two groups were created from the evaluation of psychological symptoms (in both variables, the median was used as a cut-off point). Thereby, four groups were obtained: (1) Complete Psychological Health—high life satisfaction and low psychological symptoms; (2) Incomplete Psychological Health—low life satisfaction and low psychological symptoms; (3) Incomplete Psychological Distress—high life satisfaction and marked psychological symptoms; and (4) Complete Psychological Distress—low life satisfaction and marked psychological symptoms (Table 3).

Boys were found to be significantly more frequent in the group Complete Psychological Health (45.5% boys vs. 27.7% girls), and girls were more frequent in the group with Complete Psychological Distress (41.6% girls vs. 20.8% boys; χ^2^ = 283.362; (df (3) *p* < 0.001)). In contrast, younger pupils were found to be significantly more frequent in the group with Complete Psychological *Health* (50.6% younger vs. 30.0% older), and older pupils were more frequent in the group with Complete Psychological Distress (37.9 % older vs. 18.2% younger) (χ^2^ = 246.896 (df (3) *p* < 0.001)). Furthermore, the complete analysis of the four conditions carried out during a previous study highlighted that the percentage of girls reporting symptoms was higher than that of boys, even when they reported high life satisfaction, and the percentage of boys reporting no symptoms was higher than the percentage of girls, even when they reported low life satisfaction; younger pupils more often report being satisfied with life whether or not they reported severe symptoms, and older pupils more often reported low life satisfaction whether or not they reported psychological symptoms [35,36].

Table 4 analyzes the situation of pupils in the four situations described according to the changes they felt in their relationships within various areas of their life (at school, in the family, with friends, with themselves, and in life in general). It is also clear that pupils in the most favorable quadrant (Complete Psychological Health/high life satisfaction and reduced psychological symptoms) reported less often that their life situation became worse within the diverse domains. The opposite happened with the least favorable situation (Complete Psychological Distress/low life satisfaction and pronounced psychological symptoms), in which pupils more often indicated that their life became worse within the diverse domains. It is interesting to note that perceiving their lives as “staying the same” was sometimes close to a least favorable situation, and for others, to a more favorable situation, raising the question of what exactly “staying the same” means.

### 3.1. Characteristics of the Participants According to Their Perceptions of Life Changes after the COVID-19 Pandemic

Three groups were formed to study the group differences according to the perception of changes in daily life due to the COVID-19 pandemic: life became worse, life remained the same, and life became better. Comparisons between the groups were made separately for the children and pre-adolescents (second cycle of primary education) and adolescents (lower and upper secondary education).

For children and pre-adolescents, the first group, “Better Life”, grouped the 235 students who reported perceiving that their daily life had changed positively with the COVID-19 pandemic. The second group, “Life the Same”, comprised 780 students who reported that their lives were unchanged by the COVID-19 pandemic. Finally, the third group, “Life Worse”, comprised 101 students who reported perceiving negative changes in their daily lives with the COVID-19 pandemic.

For adolescents, the first group, “Better Life”, grouped 549 students who reported perceiving that their daily lives had changed positively with the COVID-19 pandemic. The second group, “Life the Same”, comprised 2129 students who reported that their lives did not change due to the COVID-19 pandemic. Finally, the third group, “Life Worse”, comprised 387 students who reported perceiving negative changes in their daily lives with the COVID-19 pandemic. One-factor analysis of variance was performed to compare the groups at each developmental stage. As mentioned, DASS and PYD assessments were not used in children/pre-adolescents.

#### 3.1.1. Children and Pre-Adolescents

Table 5 shows the mean values and standard deviations in the three groups for each of the dependent variables analyzed and the analysis of variance results. 

The comparisons between the three groups of students in the second cycle of primary education, through the analysis of variance, showed statistically significant differences in most of the variables studied (Table 5). Multiple comparisons between groups, using Tukey’s method, evidenced that children/pre-adolescents with a better life reported higher levels of life satisfaction and wellbeing compared to children/pre-adolescents with life the same who, in turn, showed higher levels of life satisfaction and wellbeing compared to children/pre-adolescents with a worse life. In turn, these children/pre-adolescents reported higher levels of psychological symptoms when compared to children/pre-adolescents with a better or the same life (Table 5).

Comparisons between groups in the individual factors also evidenced the existence of statistically significant differences in the dimensions relating to optimism [F (2; 1107) = 125.85, *p* < 0.001], emotional control [F (2; 1106) = 33.92, *p* < 0.001], resilience/resistance [F (2; 1106) = 38.59, *p* < 0.001], confidence [F (2; 1101) = 42.21, *p* < 0.001], curiosity [F (2; 1107) = 16.58, *p* < 0.001], sociability [F (2; 1100) = 52.82, *p* < 0.001], persistence/perseverance [F (2; 1106) = 8.06, *p* < 0.001], creativity [F (2; 1102) = 18.91, *p* < 0.001], energy [F (2; 1103) = 41.36, *p* < 0.001], cooperation [F (2; 1104) = 8.84, *p* < 0.001] and self-control [F (2; 1103) = 17.94, *p* < 0.001]. 

Multiple comparisons between groups using Tukey’s method showed that children and pre-adolescents with a better life reported higher levels of optimism, emotional control and energy compared to children/pre-adolescents with the same life who, in turn, showed higher levels in these variables compared to children/pre-adolescents with a worse life. On the other hand, children/pre-adolescents with a better and the same life reported higher levels of resilience, confidence, curiosity, sociability, persistence/perseverance, creativity, cooperation and self-control compared to children/pre-adolescents with a worse life (Table 5).

When comparing the groups for school-related contextual factors, the results obtained showed the existence of statistically significant differences in the feeling of belonging to school [F (2; 1100) = 65.34, *p* < 0.001], bullying behaviors [F (2; 1103) = 43.82, *p* < 0.001], and relationships with teachers [F (2; 1104) = 3.35, *p* < 0.05]. Furthermore, post hoc comparisons using Tukey’s method found that children/pre-adolescents with a better or the same life reported a greater sense of belonging to the school, while children/pre-adolescents with a worse life reported higher levels of bullying (Table 5).

The groups were further significantly different in the number of hours of sleep [F (2; 1091) = 12.94, *p* < 0.001] and exposure to screens [F (2; 1101) = 7.32, *p* < 0.001]. Again, in multiple comparisons between groups, children/pre-adolescents with a worse life reported fewer sleep hours and more screen exposure. No statistically significant differences were found in physical activity practice (Table 5).

#### 3.1.2. Adolescents

Concerning the adolescent group, statistically significant differences were obtained in life satisfaction [F (2; 3056) = 436.4, *p* < 0.001], wellbeing [F (2; 3060) = 319.3, *p* < 0.001] and psychological symptoms [F (2; 3053) = 358.2, *p* < 0.001]. Similarly to the results obtained with children/pre-adolescents, multiple comparisons between groups using the Tukey method showed that adolescents with a better life reported higher levels of life satisfaction and wellbeing when compared to adolescents with the same life who, in turn, showed higher levels of life satisfaction and wellbeing when compared to adolescents with a worse life. In turn, these adolescents reported higher levels of psychological symptoms when compared to adolescents with a better or the same life (Table 6).

Comparisons between the groups in the factors related to individual features also evidenced the existence of statistically significant differences in the dimensions related to optimism [F (2; 3055) = 487.8, *p* < 0.001], emotional control [F (2; 3033) = 164.7, *p* < 0.001], resilience/resistance [F (2; 3035) = 210.3, *p* < 0.001], confidence [F (2; 3036) = 144.5, *p* < 0.001], curiosity [F (2; 3032) = 54.5, *p* < 0.001], sociability [F (2; 3030) = 136.5, *p* < 0.001], persistence/perseverance [F (2; 3038) = 95.4, *p* < 0.001], creativity [F (2; 3034) = 55.7, *p* < 0.001], energy [F (2; 3038) = 242.3, *p* < 0.001], cooperation [F (2; 3040) = 20.7, *p* < 0.001] and self-control [F (2; 3035) = 45.1, *p* < 0.001]. In addition, multiple comparisons between groups using Tukey’s method showed that adolescents with a better life reported higher levels in all of these dimensions when compared to adolescents with the same life who, in turn, also showed higher levels in these variables compared to adolescents with a worse life (Table 6).

When studying the contextual factors related to school, the results obtained showed the existence of statistically significant differences in the feeling of belonging to school [F (2; 3040) = 239.4, *p* < 0.001], bullying behaviors [F (2; 3033) = 35.7, *p* < 0.001], and relationships with teachers [F (2; 3034) = 19.8, *p* < 0.05]. Furthermore, in post hoc comparisons, using Tukey’s method, it was possible to verify that adolescents with a better life reported a greater sense of belonging to school compared to adolescents with the same life, who reported higher mean levels of belonging to school compared to adolescents with a worse life. In addition, adolescents with a worse life reported higher mean levels of bullying and difficulties in relationships with teachers compared to adolescents with a better or the same life (Table 6).

The groups further differed statistically significantly in the number of hours of physical activity [F (2; 3025) = 44.1, *p* < 0.001], sleep [F (2; 3008) = 95.8, *p* < 0.001] and exposure to screens [F (2; 3017) = 32.8, *p* < 0.001]. Again, in multiple comparisons between groups, adolescents with a worse life reported more hours of screen exposure than adolescents with a better or the same life. In addition, adolescents with a better life reported more hours of physical activity and more sleep than adolescents with the same life, who had higher levels of these variables than those with a worse life (Table 6).

Additionally, comparisons between the groups in psychopathological symptoms evidenced the existence of statistically significant differences in all dimensions: stress [F (2; 3011) = 274.5, *p* < 0.001], depression [F (2; 3011) = 482, *p* < 0.001], anxiety [F (2; 3011) = 255, *p* < 0.001], and in the total score [F (2; 3011) = 409.8, *p* < 0.001]. The post hoc comparisons revealed that adolescents in the group whose living conditions worsened due to the pandemic reported higher levels of psychopathological symptomatology compared to the group that maintained their living conditions. Conversely, the latter group reported higher levels of symptomatology than the group whose living conditions improved during the pandemic (Table 6).

Finally, in the dimensions of positive development, statistically, significant differences were also obtained in all dimensions: competence [F (2; 3001) = 409.8, *p* < 0.001], confidence [F (2; 3014) = 409.8, *p* < 0.001] and connectedness [F (2; 3009) = 409.8, *p* < 0.001], with adolescents in the group with improved living conditions reporting higher levels in the dimensions of positive development compared to the group that maintained living conditions, which, in turn, showed higher levels of positive development compared to the group with worsening living conditions due to the pandemic (Table 7).

## 4. Discussion

The study’s results supported the hypothesis that the dual model [23,24,25] could effectively aid in comprehending the distribution of students across various scenarios based on their levels of life satisfaction and psychological symptoms of distress. Through this model, it was found, as observed in previous studies, that, overall, girls presented a more disadvantageous situation from the point of view of their psychological distress [1,2,28,35,36,37,38,39,40].

As observed in previous studies, we also found that older students presented a more disadvantageous situation concerning their psychological distress [28,35,36].

Interestingly, the results reinforce that, globally, reporting symptoms is more frequent among girls, even when they report high life satisfaction, and reporting no symptoms is more frequent among boys, even when they report low life satisfaction; also, the youngest are the ones who most frequently report being satisfied with life, with or without marked symptoms. Conversely, the oldest most frequently report low life satisfaction, whether or not they indicate psychological symptoms. 

This fact points to the need, already mentioned by the original authors [23,24,25], to take into consideration not only the extreme situations of “complete health” and “complete distress” but also the situations in which only one of the conditions occurs (low psychological symptoms and high life satisfaction or pronounced psychological symptoms with low life satisfaction).

After accounting for other psychological wellbeing indicators, such as the perceived impact of COVID-19 on students’ lives, we observed that the previously identified pattern persisted. Specifically, students in a state of complete psychological health, characterized by high life satisfaction and reduced psychological symptoms, were more likely to report an improvement or stability in various personal and social aspects of their lives following the pandemic. In contrast, students in a state of complete psychological distress, marked by low life satisfaction and pronounced psychological symptoms, were more vulnerable and reported a decline in these aspects of their lives after the pandemic.

As also suggested by the authors and hypothesized, there is a need to consider situations in which only one condition is present (low psychological symptoms and high life satisfaction or high psychological symptoms with low life satisfaction) and to be alert to the differential position of gender across grade levels. These data suggest that throughout schooling, symptoms of psychological distress (even when there is good life satisfaction) are mainly accentuated in girls, while in boys, there is a decrease in life satisfaction, even when psychological symptoms are reduced. 

When examining the impact of the COVID-19 pandemic on young people’s daily lives, considering individual and contextual factors, a consistent pattern emerged regarding life satisfaction and psychological wellbeing, irrespective of the level of education. Specifically, all youth who perceived a decline in their daily lives due to the pandemic reported lower levels of life satisfaction and wellbeing than those who perceived no change or improvement in their daily lives. This finding was consistent with previous studies [41,42,43].

A similar pattern, also independent of the level of education, was obtained when the differences between the groups for psychopathological symptoms were studied, with young people with perceived worsening of their lives due to the pandemic reporting higher levels of psychological symptoms and bullying. Similar results were obtained by Branquinho and colleagues [8], Júnior and colleagues [17], Kecojevic and colleagues [18], and Singh and colleagues [19], showing that, regardless of age group, the pandemic had a significant impact on the psychological health of young people [44,45].

The differences between the groups in routines related to the duration of exposure to screens, the practice of physical activity and the number of hours of sleep during the week showed a differentiated pattern, with pupils with a perception of aggravation of their lives by the pandemic reporting spending a more significant number of hours on screens. In the case of physical activity and sleep hours, differences were only found for older pupils, where a perception of an improvement in their lives due to the pandemic was associated with more sleep hours and physical activity during the week. Once again, the results obtained were generally in line with the literature in this area [12,17]. 

Within the scope of socio-emotional competencies, in children and pre-adolescents (second cycle), results differentiated the pupils with a perception of aggravation of their lives from the other two groups (same, better lives) that reported higher mean levels in all dimensions of socio-emotional competencies. In contrast, in the adolescent groups (lower and upper education), these competencies differentiated the three groups (better, same and worse life), decreasing according to the perception of changes in their lives with the pandemic. 

In the adolescent groups (lower and upper education), the PYD subscale “competence” differentiated the pupils with a perception of improvement from the remaining groups. The same analysis showed that students with a perception of improvement, regardless of their level of education, had higher levels of life satisfaction. Once again, the socio-emotional skills were shown to discriminate, through different characteristics, the pupils with a perception of improvement in their lives: in children and pre-adolescent groups (second cycle), optimism and the persistence/perseverance; in the adolescent groups (lower and upper education), confidence, emotional control, sociability, creativity and sense of belonging to the school, in addition to optimism. 

The present study has some limitations that should be taken into account. It is a self-report study with a cross-sectional design, which does not allow causal inferences to be made. Despite these limitations, the participant selection was randomized and stratified by region and level of education, with a high number of participants.

## 5. Conclusions

This quadripartite model based on the dual factor model [23,24,25] allowed us to take a deeper look at the evolution of life satisfaction, perceptions of psychological symptoms of distress, and another look at gender and educational level differences. A four-scenario perspective provided relevant insight into the worsening of adolescents’ psychological health after the pandemic, accentuated the gender differences already identified, and provided a more fine-grained warning of gender and educational level differences. This study has already influenced the recommendations to the Ministry of Education and the prevention and promotion measures underway [3]. For example, train teachers to be aware of adolescents internalizing and externalizing behaviors and potential gender differences regarding reporting or not reporting psychological problems and feeling or not feeling satisfied with life. Additionally, to better help pupils overcome social and emotional difficulties, increase the collaboration between school psychologists and teachers so that they can be partners in meeting adolescents’ socio-emotional needs. Finally, increase the collaboration among principals, school psychologists and teachers so that broad “whole school” approaches can be considered. 

An analysis of the perception of the impact of COVID-19 in several areas of children’s, pre-adolescents’ and adolescents’ lives highlights that lesser impact is associated with better personal and social performance. What we do not know from this study design is whether more prepared children, pre-adolescents and adolescents feel less of an impact from the adverse situations or if not perceiving a strong negative impact makes them feel better.

Nevertheless, the results reinforce the importance of preventing psychological distress and promoting adolescents’ psychological health and wellbeing in school settings by promoting personal assets and social support (teachers, peers, family) and if needed, professional mental health help.

## Figures and Tables

**Table 1:** Distribution of participants by gender, grade/school year and cycle/level of education: frequencies and percentages.

		%	N
Gender	Boys	47.8	2054
	Girls	52.2	2241
Grade/school year	5th grade	13.4%	589
	6th grade	13.6%	596
	7th grade	10.8%	473
	8th grade	12.2%	537
	9th grade	11.9%	521
	10th grade	10.1%	443
	11th grade	16.8%	739
	12th grade	11.2%	490
Cycle/level of education	2nd cycle	27.2	1209
	Lower and upper secondary education	72.8	3235

Note: N = number of people in the population under study.

**Table 2:** Measures and variables under study.

Variables	Categories/Items	Minimum and Maximum
Gender	Boy	
	Girl	
Age	Continuous variable	Min = 9 e Max = 18
School Year	5th year to 12th year	Min = 5 e Max = 12
Cycle/Level of Education	2nd cycle	
	Lower and upper secondary education	
Cantril–Satisfaction with life (HBSC) [26]	Ladder of 11 rungs: “The top of the ladder is ‘10’ and represents the best possible life for you. The bottom of the ladder is ‘0’ and represents the worst possible life for you. Right now, where do you think you are situated on the ladder?”.	0 = worst possible life to 10 = best possible life
HBSC symptoms of psychological distress (HBSC) [27,28]	Sadness	1 to 5—Rarely or never to almost every day
	Irritability	
	Nervousness	
	Difficulty in falling asleep	
	Extreme sadness	
COVID-19 [3]	My life at school became…	worse; the same; better
	My family life has become…	
	My life with my friends has become…	
	My life with myself was…	
My life is … [3]	… overall better than other people my age	worse; the same; better
	…, in general, the same as other people my age	
	… overall worse than other people my age	
WHO5 Quality of life [29]	How you’ve been feeling for the last 2 weeks.	never to all the time
Physical Activity (HBSC) [27,28]	In the past 7 days, how many days did you engage in physical activity for at least 60 minutes?	0 to 7 days
Sleep (HBSC) [27,28]	In general, how many hours do you sleep each night?	0 to 10 or more hours
Screen time (HBSC) [27,28]	In general, how many hours do you spend each day in front of a screen (TV, mobile phone, computer, tablet)?	0 to 10 or more hours
SSES (Socio emotional skills) [30] Optimism		0 = lower; 32 = higher
Emotional control		0 = lower; 32 = higher
Resilience/Resilience		0 = lower; 32 = higher
Confidence		0 = lower; 32 = higher
Curiosity		0 = lower; 32 = higher
Sociability		0 = lower; 32 = higher
Persistence		0 = lower; 32 = higher
Creativity		0 = lower; 32 = higher
Energy		0 = lower; 32 = higher
Cooperation		0 = lower; 32 = higher
Self-control		0 = lower; 32 = higher
DASS [31,32]		
Stress		0 = lower; 21 = higher
Anxiety		0 = lower; 21 = higher
Depression		0 = lower; 21 = higher
Total		0 = lower; 63 = higher
PYD- Positive Development [33,34]		
Competence		0 = lower; 24 = higher
Confidence		0 = lower; 24 = higher
Connection		0 = lower; 32 = higher
Belonging to school		0 = lower; 24 = higher
Bullying		0 = lower; 12 = higher
Relationship with teachers		0 = lower; 9 = higher

Note: PYD–Applied to lower and upper secondary education pupils.

**Table 3:** Life satisfaction and psychological symptoms.

		%	N
Life satisfaction			
	Low life satisfaction	50.3	2223
	High life satisfaction	49.7	2198
Psychological symptoms			
	Reduced psychological symptoms	53.4	2346
	Marked psychological symptoms	46.6	2049
Life satisfaction and psychological symptoms			
	Incomplete Psychological Health	17.7	778
	Complete Psychological Distress	32.6	1430
	Complete Psychological Health	35.6	1562
	Incomplete Psychological Distress	14.1	618

Note: N = number of people in the population under study.

**Table 4:** Associations of psychological symptoms and life satisfaction with life changes at school, family life, life with friends, with oneself, and in general after the COVID-19 pandemic.

			Psychological Symptoms			
	χ^2^		Reduced		Pronounced	
School life after the COVID-19 pandemic	94.885 *** df (12)	Life satisfaction	N	%	N	%
Much worse		Low	49	13.3%	172	46.7%
		High	98	26.6%	49	13.3%
Worse		Low	159	14.8%	399	37.2%
		High	364	33.9%	151	14.1%
Same		Low	413	20.8%	583	29.4%
		High	714	36%	275	13.9%
Better		Low	86	16%	175	32.5%
		High	189	35.1%	88	16.4%
Much better		Low	37	15.9%	45	19.3%
		High	117	50.2%	34	14.6%
			Psychological symptoms			
	χ^2^		reduced		pronounced	
Family life after the COVID-19 pandemic	149.060 *** df (12)	Life satisfaction	N	%	N	%
Much worse		Low	23	10.6%	117	53.9%
		High	49	22.6%	28	12.9%
Worse		Low	102	15.6%	275	42.2%
		High	187	28.7%	88	13.5%
Same		Low	459	19.4%	739	31.2%
		High	867	36.6%	306	12.9%
Better		Low	102	18.6%	170	31%
		High	188	34.2%	89	16.2%
Much better		Low	57	14.3%	68	17.1%
		High	186	46.7%	87	21.9%
			Psychological symptoms			
	χ^2^		reduced		pronounced	
Life with friends after the COVID-19 pandemic	100.523 *** df (12)	Life satisfaction	N	%	N	%
Much worse		Low	26	13.1%	103	52%
		High	42	21.2%	27	13.6%
Worse		Low	99	13.8%	271	37.8%
		High	244	34.1%	102	14.2%
Same		Low	394	19.1%	633	30.6%
		High	776	37.5%	264	12.8%
Better		Low	162	21.1%	258	33.6%
		High	225	29.3%	123	16%
Much better		Low	60	14.2%	101	23.8%
		High	186	43.9%	77	18.2%
			Psychological symptoms			
	χ^2^		reduced		pronounced	
Life with myself	506.607 *** df (12)	Life satisfaction	N	%	N	%
Much worse		Low	32	7.7%	278	67.3%
		High	51	12.3%	52	12.6%
Worse		Low	103	13.4%	372	48.6%
		High	173	22.6%	118	15.4%
Same		Low	397	21.1%	465	24.7%
		High	786	41.7%	235	12.5%
Better		Low	1260	20.9%	162	26.9%
		High	212	35.2%	102	16.9%
Much better		Low	72	14.7%	82	16.7%
		High	252	51.4%	84	17.1%
			Psychological symptoms			
	χ^2^		reduced		pronounced	
My life is, in general, becoming…	780.131 *** df (6)	Life satisfaction	N	%	N	%
		Low	106	13.6%	90	*11.5%*
Better		High	454	58.1%	132	16.9%
		Low	592	20.5%	878	*30.4%*
Same		High	994	34.4%	428	14.8%
		Low	38	7.8%	395	81.3%
Worse		High	22	4.5%	31	6.4%

*** *p* ≤ 0.001.

**Table 5:** Comparisons between groups, according to the evaluation of current life, in the second cycle of primary education.

	Better Life (*n* = 235)		Life the Same (*n* = 780)		Worse Life (*n* = 101)		F
	M	SD	M	SD	M	SD	
Satisfaction with life	8.73	1.51	8.10	1.53	5.68	2.34	129.79 *** ^M > I > P^
Wellbeing	19.2	4.58	17.49	4.45	11.98	4.89	90.68 *** ^M > I > P^
Psychological symptoms	4.29	4.32	5.19	4.38	11.75	5.68	106.62 *** ^P > M, I^
Optimism	3.16	0.68	2.92	0.62	1.94	0.80	125.85 *** ^M > I > P^
Emotional control	2.35	0.73	2.18	0.66	1.68	0.69	33.92 *** ^M > I > P^
Resilience/Resilience	2.21	0.80	2.05	0.75	1.42	0.86	38.59 *** ^M, I > P^
Confidence	2.57	0.72	2.46	0.68	1.83	0.80	42.21 *** ^M, I > P^
Curiosity	3.09	0.69	2.96	0.62	2.65	0.75	16.58 *** ^M, I > P^
Sociability	2.98	0.66	2.80	0.64	2.18	0.79	52.82 *** ^M, I > P^
Persistence/Perseverance	2.86	0.71	2.75	0.66	2.54	0.75	8.06 *** ^M, I > P^
Creativity	2.91	0.69	2.75	0.66	2.41	0.81	18.91 *** ^M, I > P^
Energy	2.86	0.67	2.61	0.68	2.11	0.78	41.36 *** ^M > I > P^
Cooperation	3.15	0.68	3.06	0.59	2.84	0.67	8.84 *** ^M, I > P^
Self-control	2.71	0.74	2.58	0.61	2.24	0.81	17.94 *** ^M, I > P^
Sense of belonging	2.75	0.50	2.63	0.50	2.07	0.60	65.34 *** ^M, I > P^
Bullying	0.41	0.64	0.42	0.60	1.04	0.84	43.82 *** ^P > M, I^
Teacher relations	2.40	0.82	2.51	0.71	2.35	0.69	3.55 *
PA practice	3.88	2.01	3.58	1.88	3.62	2.09	2.09
Hours of sleep	8.48	1.19	8.40	1.12	7.80	1.55	12.94 *** ^M, I > P^
Screen time	3.52	2.51	3.60	2.33	4.54	2.96	7.32 *** ^P > M, I^

* *p* ≤ 0.05; *** *p* ≤ 0.001. Note: M = better life; I = life the same; P = worse life.

**Table 6:** Comparisons between groups, according to the assessment of current life, in lower secondary school students and upper secondary school students.

	Better Life (*n* = 549)		Life the Same (*n* = 2129)		Worst Life (*n* = 387)		F
	M	SD	M	SD	M	SD	
Satisfaction with life	8.2	1.5	7.1	1.61	5.0	1.9	436.4 *** ^M > I > P^
Wellbeing	17.2	4.7	14.4	4.9	9.2	4.5	319.3 *** ^M > I > P^
Psychological symptoms	4.6	4.4	6.9	4.89	12.9	4.8	358.2 *** ^P > I > M^
Optimism	3.1	0.65	2.6	0.71	1.7	0.66	487.8 *** ^M > I > P^
Emotional control	2.5	0.74	2.1	0.73	1.6	0.76	164.7 *** ^M > I > P^
Resilience/Resilience	2.4	0.86	1.9	0.83	1.3	0.77	210.3 *** ^M > I > P^
Confidence	2.3	0.76	2.2	0.69	1.6	0.75	144.5 *** ^M > I > P^
Curiosity	2.9	0.8	2.7	0.60	2.5	0.74	54.5 *** ^M > I > P^
Sociability	2.7	0.72	2.4	0.71	1.9	0.76	136.5 *** ^M > I > P^
Persistence/Perseverance	2.9	0.9	2.6	0.64	2.2	0.82	95.4 *** ^M > I > P^
Creativity	2.8	0.68	2.5	0.62	2.4	0.73	55.7 *** ^M > I > P^
Energy	2.7	0.73	2.3	0.67	1.7	0.71	242.3 *** ^M > I > P^
Cooperation	3.1	0.62	3.0	0.57	2.9	0.64	20.7 *** ^M > I > P^
Self-control	2.7	0.69	2.6	0.62	2.3	0.74	45.1 *** ^M > I > P^
Sense of belonging	2.7	0.52	2.5	0.49	1.9	0.58	239.4 *** ^M > I > P^
Bullying	0.24	0.49	0.26	0.49	0.49	0.67	35.7 *** ^P > I, M^
Teacher relations	2.4	0.79	2.4	0.75	2.1	0.84	19.8 *** ^P > I, M^
PA practice	3.8	2.1	3.1	1.8	2.8	1.8	44.1 *** ^M > I > P^
Hours of sleep	7.8	1.1	7.8	1.2	6.8	1.3	95.8 *** ^M > I > P^
Screen time	4.8	2.5	4.9	2.4	5.9	2.7	32.8 *** ^P > I, M^

*** *p* ≤ 0.001. Note: M = better life; I = life the same; P = worse life.

**Table 7:** Comparisons between groups, according to the assessment of current life in lower secondary and upper secondary school students.

	Better Life (*n* = 549)		Life the Same (*n* = 2129)		Worse Life (*n* = 387)		F
	M	SD	M	SD	M	SD	
DASS							
Stress	3.9	4.0	5.5	4.4	10.5	5.1	274.5 *** ^P > I > M^
Depression	2.6	3.5	4.7	4.3	11.2	5.3	482.0 *** ^P > I > M^
Anxiety	2.6	3.5	3.9	3.9	8.5	5.3	255.0 *** ^P > I > M^
Total DASS	9.1	10.1	14.1	11.3	30.1	13.9	409.8 *** ^P > I > M^
PYD							
Competence	16.4	4.7	13.4	4.2	9.6	4.7	272.2 *** ^M > I > P^
Confidence	18.1	4.63	14.4	5.0	8.6	5.5	401.4 *** ^M > I > P^
Connectedness	22.7	5.75	20.4	5.3	14.9	5.3	238.8 *** ^M > I > P^

*** *p* ≤ 0.001. Note: M = better life; I = life the same; P = worse life.

## Data Availability

The data presented in this study are available on request from the corresponding author.
